# Identifying and prioritizing industry-level competitiveness factors: evidence from pharmaceutical market

**DOI:** 10.1186/2008-2231-22-35

**Published:** 2014-04-03

**Authors:** Hosein Shabaninejad, Gholamhossein Mehralian, Arash Rashidian, Ahmad Baratimarnani, Hamid Reza Rasekh

**Affiliations:** 1 Department of Health Services Management, School of Health Management and Information Sciences, Iran University of Medical Sciences, Rashidiasemi st, Valiasr st, Vanak sq., P.O.Box: 1995614111, Tehran, Iran; 2 Department of Pharmacoeconomics and Pharma Management, School of Pharmacy, Shahid Beheshti University of Medical Sciences, Tehran, Iran; 3 Department of Health Management and Economics, School of Public Health & Knowledge Utilization Research Center, Tehran University of Medical Sciences, Tehran, Iran

**Keywords:** Competitiveness, Pharmaceutical industry, Key factors, Human capital, Iran

## Abstract

**Background:**

Pharmaceutical industry is knowledge-intensive and highly globalized, in both developed and developing countries. On the other hand, if companies want to survive, they should be able to compete well in both domestic and international markets. The main purpose of this paper is therefore to develop and prioritize key factors affecting companies’ competitiveness in pharmaceutical industry. Based on an extensive literature review, a valid and reliable questionnaire was designed, which was later filled up by participants from the industry. To prioritize the key factors, we used the Technique for Order Preference by Similarity to Ideal Solution (TOPSIS).

**Results:**

The results revealed that human capital and macro-level policies were two key factors placed at the highest rank in respect of their effects on the competitiveness considering the industry-level in pharmaceutical area.

**Conclusion:**

This study provides fundamental evidence for policymakers and managers in pharma context to enable them formulating better polices to be proactively competitive and responsive to the markets’ needs.

## Background

Pharmaceutical industry has changed tremendously in recent years [1]. The importance of big changes in pharmaceutical industry should be considered due to intensity in Research and Development (R&D) activities [2,3], uncertainty in drug development process, lack of new products [4], rapid integration [5], rapid development of generic markets [6] and finally increased global competition and technological advances [7]. Moreover, some unique characteristics such as high-regulated setting, the long development process, risky and high level of cost in research phase [8] distinguish the pharmaceutical industry from other industries.

While competition is increasing tremendously, there is an immediate need for the pharmaceutical companies to behave in a good, sharp and speedy manner. With respect to new competitive environment, it is more important to consider the factors affecting competitiveness of pharmaceutical industries in the internationalized and globalized market [9]. This study tries to identify and prioritize key factors that affecting pharmaceutical competitiveness at the industry level, based on pharmaceutical managers’ perspective. The rest of the paper proceeds as follows. The first section presents an overview of pharmaceutical industry in the world and Iran. The next section describes the research methodology, followed by the result of the study. The final section presents discussion and concludes by considering the practical implications and limitations of the study.

### Global pharmaceutical industry

Throughout the last decades, the global pharmaceutical industry has been one of the most successful and profitable industries [10], but, as mentioned, due to dynamic forces in the competitive as well as regulatory environment, the conditions of the industry have changed. Given to the strong dependency on innovation, some issues such as the high risks in R&D as well as supply chain [11], cause to decrease the attractiveness of pharmaceutical industry [12] compare to other industries.

While expenditure on R&D has increased steadily over the last decade, the number of New Molecular Entities (NMEs) being brought to the market has decreased. It is important since further development depends on the number of new medicines launched from which the profit serves to fund [12], however drug development new medicine and marketing is a costly, time consuming and risky process. Based on studies, an average cost of approximately $800 million is the cost of bringing a new drug to the market [13,14]. Moreover, it is estimated that an average of 12 years would have been passed from the synthesis of the new active pharmaceutical materials to launch a new drug to the market [15]. Thereby, on average, out of every 10,000 ingredients synthesized in the laboratories, only one or two will successfully pass all the steps to become marketable medicines [16]. Meanwhile, international competitiveness is becoming important for the pharmaceutical sector more. Increased competitiveness and the changing structure of competitors impact the strategic direction of the world pharmaceutical companies in world [9]. On the other hand, companies try to increase the profitability of all phases of the value chain from primary discovery research to production phase and logistics as well as sales and marketing phases [17].

Though, managing pharmaceutical industry effectively and efficiently is vital in developing countries for their health system and economy [18], according to the lack of economic motivations and low capacity of the government for covering the costs of innovative drugs in emerging markets, usually the pharmaceutical sector doesn’t invest on novel medicines, thereby innovations are limited in such countries.

### Iranian pharmaceutical market

Iran pharmaceutical industry experienced the average growth rate of 28.38% over the last 10 years. The value of medicine which locally manufactured is $1.639 billion, while imported products comprise $0.828 billion during the same period [19]. Moreover Iran’s pharmaceutical industry has witnessed profound changes in recent years; the producers were working in an atmosphere of ever-increasing demand, and due to lack of competition, no motives remained for marketing, sales and quality improvement of drugs [20]. Recently, the market has become more competitive as a result of foreign medicines importation; as such in 2000, there were only 53 pharmaceutical manufacturing and 12 companies importing to Iran, these figures were increased to 89 and 93, respectively [19].

## Methods

### Reliability and validity of the questionnaire

To assess the managers’ perspective about the status of key factors on competitiveness of pharmaceutical industry, a questionnaire was designed based on an extensive literature review as well as interviews with pharmaceutical experts. Reliability and validity tests were conducted on the questionnaire with multivariate measures. The validity of a tool refers to the extent which it measures what is intended to be measured [21]. To assess the acceptance of the questionnaire, 10 people involved for at least 10 years in the field of pharmaceutical practice were invited to participate in a pilot test. The participants proposed revising parts of the questionnaire. At the end, all participants expressed high agreement with the appropriateness of the questionnaire. The questionnaire finalized after modifying some questions accordingly. Cronbach’s alpha reliability was applied to measure the internal consistency of these multivariate scales [21]. The results showed that the Cronbach’s alpha for all dimensions was as 0.89, which indicates strong reliability for our survey instrument.

### Data collection

Data for this study have been collected using questionnaires distributed to 25 pharmaceutical firms, which were affiliated to three large pharmaceutical holding companies. To gather the viewpoints of the pharmaceutical industry`s executives, the questionnaires were sent to managers in marketing, sales, information technology (IT), finance, R&D, quality assurance and quality control departments. As Table 1 shows, most of participants have more than 10 years of job experience in the pharmaceutical industry (80 percent), and a quarter of the participants were top managers. Although, probability sampling is preferred over non-probability sampling [22], in some cases it is not feasible, practical or theoretically sensible to consider the probability of sampling. Accordingly, we chose the respondents from managers with comprehensive knowledge about pharmaceutical industry, strategy, international pharmaceutical markets, and general pharmaceutical management issues. The number of questionnaires sent out was 240; the number of returned ones was 169 resulting in a response rate of 70 percent. Three percent of the returned questionnaires were incomplete. Non response bias was checked by comparing for all constructs by ANOVA and produced no significant differences. This study was approved by Iran University of Medical Sciences ethics committee. Participants in this study were informed that; participating in this study is voluntary, they are free not to answer some questions they don’t like to answer and their biography will be treated as confidential and will not be published. Moreover participants in this study provided informed consent for publication of this work.

**Table 1:** Sample characteristics

**Construct**	**Classification**	**Number**	**Percentage**
Position	Managerial	41	24.3
	Financing	8	4.7
	Production	32	18.9
	R&D	28	16.6
	Marketing	29	17.2
	Human resource	2	1.2
	Regulatory	9	5.3
	QC	20	11.8
Job experience	Under 3 years	11	6.6
	4-9 years	22	13
	10-15 years	68	40.2
	Up 10 years	68	40.2

**R&D:** Research and Development **QC:** Quality control.

The pharmaceutical industry competitiveness factors prioritization questionnaire is included as Additional file 1.

### Factor analysis

Factor analysis is a procedure that relies on the use of correlations between data variables [23]. In this study, each factor was individually tested for construct validity. The confirmatory factor analysis (i.e. Pearson’s principal component analysis) was tested with and without rotation (i.e. Varimax rotation with Kaiser Normalization). The conservative factor loadings of greater than 0.5 were considered at 95% level of confidence [24].

### Data analysis with Fuzzy TOPSIS

We used Fuzzy TOPSIS for analyzing the data. TOPSIS technique of solving the multi-criteria decision chooses tasks that imply full and complete information on criteria, expressed in numerical type [25]. The method is helpful for solving real problems of managerial decisions. It provides us the optimal solution or the alternative’s ranking. The TOPSIS technique would explore among the assumed choices and come upon the one closest to the ideal solution but furthest from the anti-ideal point simultaneously [26]. Justification of the method intends to adjust a different mode of finding out the ideal and anti-ideal solution through standardization of lingual features` quantifying and introducing of fuzzy numbers in description of the features for the criteria expressed by linguistic variables [27].

## Results

Considering extensive literature review, we found 150 factors affecting the competitiveness. Based upon the experts’ opinions, 44 variables were finalized as factors with impact on competitiveness of pharmaceutical industry and citations for each factor are shown in Table 2. As noted, some items (variables 37 and 38) were developed from experts’ opinions.

**Table 2:** Pharmaceutical competitiveness variables and related citations

**Variables**	**Citations**
1- Graduates with degrees in sciences relevant to pharmaceutical industry	
2- Pharmaceutical expertise employment	[28-30]
3- Pharmaceutical managers’ experience in internal and international related area	
4- Scientific research publication relevant to pharmaceutical area	
5- Clinical studies	
6- Investment in pharmaceutical high-tech	
7- GMP structure improving investment in pharmaceutical industry	[31-36]
8- Investment in pharmaceutical research & development	
9- Production process sophistication in pharmaceutical area	
10- Using information technology in pharmaceutical industry	
11- Attract capital from market in pharmaceutical industry	
12- Venture capital in pharmaceutical area	[30,31,37]
13- Foreign direct investment in pharmaceutical industry	
14- Pharmaceutical’s market approval procedures	
15- Pharmaceutical regulation	
16- Pharmaceutical standards implementation like GMP	
17- Generic system development	[31,38-44]
18- Price regulation in pharmaceutical market	
19- Pharmaceutical intellectual property right	
20- Pharmaceutical corporation tax	
21- Intensity of domestic competition in pharmaceutical market	
22- Mergers and acquisition in pharmaceutical area	[31,37,45-47]
23- Pharmaceutical import tariff	
24- Existence of major pharmaceutical MNCs branches in domestic market	
25- Privatization in pharmaceutical industry	
26- Availability of latest technologies in pharmaceutical industry	
27- Availability of specialized research and training services in pharmaceutical area	[37,48-50]
28- Quantity and quality of local supplier in pharmaceutical market	
29- Extent of cluster policy and collaboration inside clusters in pharmaceutical industry	
30- Extent of marketing in pharmaceutical market	
31- Quality of drugs	
32- NMEs production in pharmaceutical industry	[7,51-54]
33- Diversification of production in pharmaceutical market	
34- Value chain breadth in pharmaceutical industry	
35- Degree of customer orientation of pharmaceutical market	
36- Extent of staff training in pharmaceutical industry	
37- Relationship-based recruitment in pharmaceutical area	[37], (Items 37 and 38 extract from expert opinions)
38- Frequent changes in pharmaceutical industry at the management level	
39- Pharmaceutical companies’ joint venture with MNCs	
40- Pharmaceutical companies’ alliance with MNCs	[30,37]
41- Breadth of pharmaceutical international market	
42- Macro policy (export incentives, simplifying customs regulations)	
43- Country’s political situation	[55,56]
44- Relations with the countries of the region and the world	

**GMP:** Good Manufacturing Practice **MNCs:** Multi- national companies **NMEs:** New molecular entities.

Based on factor analysis, 10 items were identified as key factors affecting pharmaceutical industry’s competitiveness, and we renamed them: human capital, macro-level policies, strategy and operational effectiveness, supporting and related industries and clusters, administrative infrastructure, capacity for innovation, organizational practices, capital market infrastructure, internationalization of firms and context for strategy and rivalry. The variance explained by these factors was as 81.94 percent, and the Cronbach alpha for each construct was larger than 0.7, which indicates an acceptable degree of consistency for constructs determined in this study as the key factors affecting pharmaceutical industry’s competitiveness. The results of factor analysis and reliability of factors are shown in Table 3.

**Table 3:** Factor analysis and reliability of factors

**Key factors**	**KMO value**	**Factor loading**	**Percentage variance Explained**	**Cronbach’s alpha**
Human capital	0.57	0.78 - 0.94	0.74	0.92
Capacity for innovation	0.61	0.46 - 0.73	0.54	0.86
Capital market infrastructure	0.48	0.40 - 0.86	0.73	0.79
Administrative infrastructure	0.70	0.12 - 0.78	0.67	0.84
Context for strategy and rivalry	0.48	0.11 - 0.87	0.53	0.78
Supporting and related industries and clusters	0.61	0.46 - 0.81	0.68	0.81
Strategy and operational effectiveness	0.57	0.35 - 0.82	0.59	0.84
Organizational practices	0.54	0.46 - 0.77	0.73	0.85
Internationalization of firms	0.57	0.56 - 0.90	0.76	0.88
Macro-level policies	0.53	0.48 - 0.92	0.63	0.89

Prioritization of key factors affecting competitiveness of pharmaceutical industry with Fuzzy TOPSIS technique is shown in Table 4.

**Table 4:** Rank of fuzzy TOPSIS for competitiveness key factors

**Pharmaceutical industry’s competitiveness key factors**	**Important level**	**Distance from positive ideal**	**Distance from negative ideal**	**Key factors’ rank**
Human capital	0.67	0.56	1.15	1
Macro-level policies	0.65	0.61	1.13	2
Strategy and operational effectiveness	0.56	0.73	0.91	3
Supporting and related industries and clusters	0.54	0.76	0.91	4
Administrative infrastructure	0.54	0.74	0.87	5
Capacity for innovation	0.54	0.74	0.87	6
Organizational practices	0.49	0.86	0.82	7
Capital market infrastructure	0.48	0.89	0.82	8
Internationalization of firms	0.42	0.95	0.70	9
Context for strategy and rivalry	0.27	1.17	0.43	10

Fuzzy TOPSIS’s results show the human capital ranking as the first key factor affecting the competitiveness of pharmaceutical industry followed by macro-level policies and state remaining factors, respectively. Context for strategy and rivalry was ranked as the last key factor.

## Discussion

Given to complexity of the pharmaceutical industry in relation with the research, regulatory and healthcare systems, here, a set of key factors including macro-level policies, strategy and operational effectiveness, supporting and related industries and clusters, administrative infrastructure, capacity for innovation, organizational practices, capital market infrastructure, internationalization of firms, context for strategy and rivalry and above all human capital were identified. These factors provide a fairly consistent and coherent explanation about the structure of competitiveness and its determinants in pharmaceutical industry.

The results of this study show human capital with the highest rank affecting the pharmaceutical competitiveness. However, the context of study is in Iran, as a typical of middle-income country, the importance of human capital on productivity and its direct impact on competitiveness were shown by studies [29,35]. On the other hand, pharmaceutical industry as a science-driven and high-tech industry [12], depend highly on skilled human resources [3]. Regarding to the importance of R&D on success of pharmaceutical companies [3,57], as a long-term and risky procedure [4,13,14], they highly rely on good quality of science graduates. Thus, it is important to pay more attention on R&D activities in pharmaceutical sector.

The formation of pharmaceutical industry in Iran was along with the government entry in this industry and the selected structure for the industry was based on government support [19]. Thus, pharmaceutical companies’ strategies, administrative infrastructure, technologies, market size, human capital and productivity of pharmaceutical companies were affected by this structure. If Iran pharmaceutical industry wants to enter to international market, this structure should be redesigned and the factors determined in this study could be used as a baseline for defining new strategies and policies for making a competitive pharmaceutical industry.

Factors identified in this study were in turn with porter’s competitiveness framework [55]. However, in this study, the demand side of the competitiveness was not identified as a key factor affecting pharmaceutical industry’s competitiveness. In pharmaceutical industry, the demand side consists of three groups including patients, physicians and hospital boards. The ultimate consumer – the patient – usually does not have much effect on a physician’s decision about a certain prescriptive drug, since their knowledge about the respective drug is limited [12]. Moreover, due to payment of drugs cost by health insurance companies, patient normally does not carry the costs of the product. This implies that patients don’t have a key role in the demand side of pharmaceutical market.

Like other studies, innovation was identified as a key factor affecting competitiveness [14,32,34]. Considering the nature of pharmaceutical industry, producing new drugs needs high investment and using state of the art technologies, as such, if developing countries want to be competitive in pharmaceutical industry, other factors have more importance than innovation. Moreover, according to the definition of innovation in the literature [58], the results indicate that pharma companies in middle-income countries should rely on incremental innovation more than on the radical one.

### Managerial relevance

This study has some managerial applications. First, competitiveness factors constructed in this study will be the starting points for future studies at the industry level in competitiveness area. Moreover, the valid and reliable tool designed in this study can be used by other researchers to assess industry-level competitiveness in middle-income countries. Since, this study was done in Iran, the generalizability of the results of this study should be considered cautiously to other contexts.

Second, based on key factors identified, the results of this study will help the managers adopting decisions to enhance the competitiveness of the firms. Finally, competitiveness factors identified in this study will be helpful to managers considering them in their decisions and strategic planning at business level.

### Limitation of the study

As a first limitation, although we try to identify all related key factors affecting pharmaceutical industry’s competitiveness, but the result of the study should be delivered in other contexts with some modifications. On the other hand, while operationalizing of most competitiveness measures are difficult [59], the key factors proposed in this study should be a basis to determine a set of constructs, which are reasonably approximate to other context. Moreover, although our sample is representative for Iranian managers who involved in pharmaceutical companies, a larger sample could help to improve the generalizability of results.

## Conclusions

Understanding competitiveness in pharmaceutical industry is a major concern of policymakers and a major challenge to provide evidences for decision making. This study provides fundamental evidence for policymakers in pharma context to enable them formulating better polices to be proactively competitive and responsive to the markets’ needs. Moreover, this work provides a tool for governments and national agencies to assess competitiveness in pharmaceutical industry and to develop measures which can be appropriate for their context. Furthermore, it enables us to compare competitiveness status of similar countries according to their strengths and weaknesses.

## Competing interests

The authors declare that they have no competing interests.

## Authors’ contributions

HRR carried out designing and conceptual modeling of the study. AR helped in designing methodology of the study. GM performed the statistical analysis. HS developed the idea of the study and participated in designing the methodology, data gathering, data analysis and drafting the manuscript. AB helped to draft and edits the manuscript. All authors read and approved the final manuscript.

## Supplementary Material

Additional file 1 Pharmaceutical industry competitiveness factors prioritization questionnaire. Click here for file
