# Supplementary material for: Identifying and prioritizing industry-level competitiveness factors: evidence from pharmaceutical market
Source: Daru. 2014 Apr 3;22(1):35. doi: 10.1186/2008-2231-22-35 (PMC4234990; doi:10.1186/2008-2231-22-35)
Supplement: Additional file 1 — Pharmaceutical industry competitiveness factors prioritization questionnaire. [file 2008-2231-22-35-S1.pdf]

Pharmaceutical Industry Competitiveness Factors prioritization Questionnaire

This is a research project that is being done by Shahid Beheshti University of Medical Sciences (SBUMS). The purpose of this study is to identify and prioritize key factors that affect competitiveness of pharmaceutical at the industry level. Pharmaceutical industry competitiveness is the ability of the nation's pharmaceutical firms to achieve sustained success against (or compared to) foreign competitors, without protection or subsidies. The nation's firms are those whose headquarters are located in the nation.

Important notes:

Please note that no information will be released that might identify anyone and confidentiality of survey respondents is ensured.

If you do not know the answer to a question or if the question does not apply, please leave it blank.

Biographical Details (please remember that we will not identify you and that only summary values of respondants will be reported to ensure confidentiality)

Please mention you email address below if you want to receive an electronic version of the results of this research:

Your Position: top manager ☐ middle manager ☐ operational manager ☐

How many years have you worked in pharmaceutical sector:
less than 1 ☐ 1 to 5 ☐ 6 to 10 ☐ 11 to 20 ☐ more than 20 ☐

Which of the following is your major activity in pharmaceutical industry?
managerial ☐ financial ☐ manufacturing ☐
Research & Development ☐ Marketing ☐ Human Resources ☐
Regulatory ☐ Commerce ☐ Quality Control ☐ other: ----------------

How would you assess the importance of following factors on competitiveness of pharmaceutical industry	Very low	Low	Neutral	High	Very high
1- Graduates with degrees in sciences relevant to pharmaceutical industry					
2- Pharmaceutical expertise employment					
3- Pharmaceutical managers' experience in internal and international related area					
4- Scientific research publication relevant to pharmaceutical area					
5- Clinical studies					
6- Investment in pharmaceutical high-tech					
7- GMP structure improving investment in pharmaceutical industry					
8- Investment in pharmaceutical research & development					
9- Production process sophistication in pharmaceutical area					
10- Using information technology in pharmaceutical industry					
11- Attract capital from market in pharmaceutical industry					
12- Venture capital in pharmaceutical area					
13- Foreign direct investment in pharmaceutical industry					
14- Pharmaceutical's market approval procedures					
15- Pharmaceutical regulation					
16- Pharmaceutical standards implementation like GMP					
17- Generic system development					
18- Price regulation in pharmaceutical market					
19- Pharmaceutical intellectual property right					
20- Pharmaceutical corporation tax					
21- Intensity of domestic competition in pharmaceutical market					
22- Mergers and acquisition in pharmaceutical area					
23- Pharmaceutical import tariff					
24- Existence of major pharmaceutical MNCs branches in domestic market					
25- Privatization in pharmaceutical industry					
26- Availability of latest technologies in pharmaceutical industry					
27- Availability of specialized research and training services in pharmaceutical area					
28- Quantity and quality of local supplier in pharmaceutical market					
29- Extent of cluster policy and collaboration inside clusters in pharmaceutical industry					
30- Extent of marketing in pharmaceutical market					
31- Quality of drugs					
32- NMEs production in pharmaceutical industry					
33- Diversification of production in pharmaceutical market					
34- Value chain breadth in pharmaceutical industry					
35- Degree of customer orientation of pharmaceutical market					
36- Extent of staff training in pharmaceutical industry					
37- Relationship-based recruitment in pharmaceutical area					
38- Frequent changes in pharmaceutical industry at the management level					
39- Pharmaceutical companies' joint venture with MNCs					
40- Pharmaceutical companies' alliance with MNCs					
41- Breadth of pharmaceutical international market					
42- Macro policy (export incentives, simplifying customs regulations)					
43- Country's political situation					
44- Relations with the countries of the region and the world					

«Thanks you very much for completing this survey»
